# Toxicological Evaluation of Lactase Derived from Recombinant *Pichia pastoris*

**DOI:** 10.1371/journal.pone.0106470

**Published:** 2014-09-03

**Authors:** Shiying Zou, Xiaoyun He, Yifei Liu, Delong Chen, Yunbo Luo, Kunlun Huang, Wei Zhang, Wentao Xu

**Affiliations:** 1 Laboratory of Food Safety and Molecular Biology, College of Food Science and Nutritional Engineering, China Agricultural University, Beijing, People’s Republic of China; 2 The Supervision, Inspection and Testing Center of Genetically Modified Organisms, Ministry of Agriculture, Beijing, People’s Republic of China; 3 Biotechnology Research Institute, Chinese Academy of Agricultural Sciences, Beijing, People’s Republic of China; USDA-ARS, United States of America

## Abstract

A recombinant lactase was expressed in *Pichia pastoris*, resulting in enzymatic activity of 3600 U/mL in a 5 L fermenter. The lactase product was subjected to a series of toxicological tests to determine its safety for use as an *enzyme preparation* in the dairy industry. This recombinant lactase had the highest activity of all recombinant strains reported thus far. Acute oral toxicity, mutagenicity, genotoxic, and subchronic toxicity tests performed in rats and mice showed no death in any groups. The lethal dose 50% (LD_50_) based on the acute oral toxicity study is greater than 30 mL/kg body weight, which is in accordance with the 1500 L milk consumption of a 50 kg human daily. The lactase showed no mutagenic activity in the Ames test or a mouse sperm abnormality test at levels of up to 5 mg/plate and 1250 mg/kg body weight, respectively. It also showed no genetic toxicology in a bone marrow cell micronucleus test at levels of up to 1250 mg/kg body weight. A 90-day subchronic repeated toxicity study via the diet with lactase levels up to 1646 mg/kg (1000-fold greater than the mean human exposure) did not show any treatment-related significant toxicological effects on body weight, food consumption, organ weights, hematological and clinical chemistry, or histopathology compared to the control groups. This toxicological evaluation system is comprehensive and can be used in the safety evaluation of other *enzyme preparations*. The lactase showed no acute, mutagenic, genetic, or subchronic toxicity under our evaluation system.

## Introduction

Lactose is the main sugar that is present in milk and dairy products. It can improve the absorption rate of some essential trace elements such as calcium, phosphorus and magnesium [1], [2]. Lactase, also known as lactase-phlorizin hydrolase or LPH, belongs to the β-galactosidase family of enzymes. It is a glycoside hydrolase that hydrolyzes the disaccharide lactose to produce galactose and glucose [3], [4]. It has been reported that lactose can be degraded by lactase and that its digestion and absorption depend on lactase activity [2]. Lactase can be produced in the digestive systems of infants and in many but not all adults [5]. The lactase gene is expressed exclusively in the colon during fetal development and declines dramatically after weaning in most of the world’s populations [6], leading to lactose intolerance in adults [7]. More than 70% of people have lactose intolerance and cannot easily digest fresh milk and dairy products [8].

In contrast, many individuals still maintain high levels of lactase throughout life, a condition known as lactase persistence [9]. Genetic researchers have found that lactase persistence in northern Europeans is strongly associated with a single nucleotide polymorphism (SNP) located 14 kb upstream of the lactase gene: -13910*C/T [10]. Lactose can be hydrolyzed by lactase into galactose and glucose, which can be absorbed easily in the gut. Galactose is an essential component in brain tissue, and it is necessary for infant brain development [10]. Additionally, lactase plays an important role in immune tests and diagnoses [11]. Therefore, commercialized lactase is used as food additive and is added to milk to produce “low-lactose” milk products. It is usually a light brown liquid that is odorless and non-toxic. There are no reports of natural lactase producing any toxic effects in human or animals. Many microorganisms, including bacteria, molds and yeast, are often used to produce lactase, including lactic acid bacteria, *Escherichia coli*, *Aspergillus niger*, *Kluyveromyces lactis* and *Kluyveromyces fragilis*. The molecular mass of lactase in *Escherichia coli* is 520–850 kD [12]. Takatoshi reported 4 strains of yeast that can product lactase, including *Tolulopsis versatilis* M6, *Tolulopsis sphaerica* J28, *Canadida pseudotropicalis* B57 and *Cadadia psedotopicalis* A60, which produce proteins with molecular masses of 226, 233, 200 and 215 kD, respectively [12].

The production of lactase by microbial strains using traditional mutation breeding cannot satisfy the fast-growing market demands. The development of genetic engineering technology makes the large-scale industrial production of lactase possible through the introduction or modification of genes to promote characteristics such as higher enzyme activity or higher production. Domingues *et al.* reported the construction of recombinant strains using the *lac*A gene from *Aspergillus niger* and *Saccharomyces cerevisiae*, which can produce a high quantity of lactase [13]. A classical chemical mutagenesis protocol was evaluated for increasing beta-galactosidase production by probiotic bacteria to improve their potential to treat the symptoms of lactose malabsorption in humans [14].

In a previous study, the *lactase* gene (*lacm*) of *Aspergillus oryzae* was placed behind an alpha-factor signal sequence in the *P. pastoris* expression vector pPIC9, and the gene was integrated into the genome of *P. pastoris* by recombination. The lactase that was expressed in *P. pastoris* was shown to be a glycosylated protein. The expression level of lactase in recombinant *P. pastoris* was 20 mg/mL, with enzymatic activity of 13000 U/mL in a 3.7 L fermenter [15], which is the highest activity among all reported recombinant strains. Morever, this lactase expression level was much higher than that of the genetic engineering *Aspergillus oryzae* [16]. This lactase derived from recombinant microorganism needs to be evaluated in accordance with the Guideline for the Conduct of Food Safety Assessment of Foods Produced Using Recombinant-DNA microorganisms (CAC/GL 46-2003).

This study was conducted to assess the toxicological properties of lactase produced by genetically modified *P. pastoris* for use as a food additive. The toxicity of recombinant lactase was assessed by an acute oral toxicity study, a mutagenicity study in bacteria (*Salmonella Typhimurium*/mammalian microsomal enzyme test, Ames test), bone marrow cell micronucleus test, mouse sperm abnormality test, and a subchronic toxicity study (90-day feeding test in rats). These trials were conducted at The Supervision, Inspection and Testing Center of Genetically Modified Organisms, at the Ministry of Agriculture (Beijing, China), in accordance with the Chinese standard *Procedures and Methods of Toxicology Assessment for Food Safety* (GB15193-2003) and in compliance with the *OECD Good Laboratory Practice* guidelines [17], [18], [19], [20]. All animal study was approved by Animal Experimental Welfare & Ethical Inspection Committee (No.100034), The Supervision, Inspection and Testing Center of Genetically Modified Organisms, Ministry of Agriculture (Beijing, China).

## Materials and Methods

Lactase was supplied by the Biotechnology Research Institute of the Chinese Academy of Agricultural Science. The enzyme was concentrated using ultrafiltration, and then purified by anion-exchange chromatography. The purity of the protein was analyzed by SDS–PAGE and gel filtration chromatography. Finally, the purified protein had been lyophilized using a vacuum freeze-drying apparatus [21]. All trials were conducted at the SPF animal laboratory of the Center with the license number SYXK (Beijing) 2010-0036. All animals were obtained from Vital River Laboratories, Inc. (Beijing, China), with the license number SCXK (Beijing) 2012-0001, and they were acclimatized for one week in cages with 5 animals of each sex per cage. The temperature of the animal room was maintained at 22±2°C, with a relative humidity between 40% and 70% and a 12-hour light/dark cycle. The diet was provided in stainless steel cans covered by a perforated stainless steel plate to prevent spillage. The diet in the feeders was refreshed weekly. Food and water were provided *ad libitum* throughout the study. During the acclimatization period, animals were fed with the rodent basic feed made by Ke Ao Xie Li Feed Co., Ltd. (Beijing, China), with the license number SCXK (Beijing) 2009-0015. All ingredients were adjusted to meet the requirement of rats (GB 14924.3-2010) and the rodent feeds were exposed to _60_Co to keep them sterile.

### 1. Acute oral toxicity study

#### 1.1. Test material and animals

Liquid lactase with an activity of 10,000 U/mL was used in this study. Twenty 5-week-old Sprague-Dawley (SD) rats (10 male and 10 female) were involved in the study.

#### 1.2. Experimental design

After acclimatization, rats of each gender were divided into two experimental groups (5/sex/group) by body weight (BW), and there were no statistically significant differences in weight between the groups. The differences between the maximum and minimum weight in each group were less than ten percent. One group was treated with recombinant lactase (10 mL/kg·BW), and one group was treated with physiological saline as a control. The substances were given by oral gavage after fasting for 12 h [22]. To confirm the maximum tolerated dose of the lactase solution, all rats were treated three times during one day, at an interval of 4 h. The maximum tolerated dose was 30 mL/kg·BW. The animals were continuously observed for 24 h, and behavioral changes were recorded for the following 14 days, including mortality, morbidity, mental status, coat condition, skin condition, eye excretions and other noteworthy clinical signs of toxicity. Body weight was measured before treatment and 7 and 14 days post-treatment. On Day 15, the rats were killed by decapitation under anesthesia. Necropsies were performed, and gross observations were recorded [23].

### 2. Mutagenicity test in bacteria (Ames test)

#### 2.1. Materials

Lactase powder with a purity of 83.33% was used in this study. To investigate the mutagenic potential of the lactase, four histidine-requiring *Salmonella Typhimurium* mutant strains were tested: TA97, TA98, TA100 and TA102. The rat liver-derived metabolic activation system (S9) and Cofactor-II were purchased from the Chinese center for disease control and prevention (CDC). The S9 mix was prepared just before use. Without S9 addition, Nexon is the positive control for TA97, TA98 and TA102; NaN3 is the positive control for TA100. With S9 addition, 2-AF is the positive control for TA97, TA98, TA100 and TA102.

#### 2.2. Experimental design

The dosages of lactase were 5000, 2500, 1250, 625 and 312.5 µg/plate. Lactase was dissolved in distilled water at concentrations of 50, 25, 12.5, 5 and 2.5 mg/mL. The bacteria were exposed to the lactase treatments in phosphate buffered nutrient broth with or without S9 mix. The concentrations of the positive controls were set in accordance with Chinese Standard GB15193.4-2003. Distilled water was used as the negative control. The test was considered positive for mutagenicity if the number of reverse mutation colonies was at least 2-fold greater for the lactase treatments than for the negative control and if the number of colonies was affected in a dose-related fashion. Otherwise, lactase was determined to be negative for genotoxicity [22].

### 3. Genotoxicity study (bone marrow cell micronucleus test)

#### 3.1. Materials and animals

Lactase powder with a purity of 83.33% was used in this test. Sixty 10-week-old KM mice (30 male and 30 female) were used. Cyclophosphamide was used as the positive control.

#### 3.2. Experimental design

The mice were divided into 5 groups, with 6 animals of each sex per group. Three groups were chosen as experimental groups and were given lactase by oral gavage in dosages of 1250, 625 and 312.5 mg/kg·BW. The negative control group was treated with physiological saline, and positive controls were treated with cyclophosphamide (40 mg/kg·BW). All mice were treated twice, with 24 h between treatments. Six hours after the second gavage, the mice were sacrificed by cervical dislocation, and bone marrow cells were immediately collected from the femurs. The bone marrow was flushed from the femurs with fetal bovine serum, and the marrow was harvested to make smears. The smears were air-dried, fixed, and stained with 4% Giemsa. Giemsa solution can stain immature erythrocytes and erythrocytes with a micronucleus so that they turn blue, whereas mature erythrocytes without micronuclei are stained pinkish orange [24]. One thousand polychromatic erythrocytes were recorded per mouse by observation under a Leica DM2500 microscope. Cells were considered to be micronucleated if they contained defined chromatin corpuscles less than one-third the diameter of a normal nucleus [25]. The frequency of micronucleated cells was calculated by counting them and dividing by the total number of polychromatic erythrocytes.

### 4. Mouse sperm abnormality test

#### 4.1. Materials and animals

Lactase powder with purity of 83.33% was used in this test. Thirty 8-week-old male KM mice were used in this test. Cyclophosphamide was used as the positive control.

#### 4.2. Experimental design

Grouping and dosing were similar to the bone marrow cell micronucleus test. Lactase was given by oral gavage for five days, and the mice were fed with rodent basic feed for 30 days. On day 35, the mice were killed by cervical dislocation, and smears of sperm from the epididymis were fixed with methanol and stained with Eosin Y [26]. One thousand sperm cells were counted, and the percentage of abnormal sperm was recorded for each mouse. The percentage of abnormal sperm was calculated as the number of abnormal cells number divided by total sperm cell count. The sperm was analyzed by microscopy (Leica, DM2500, USA), and the classification proposed by Krzanowska was used [27].

### 5. Subchronic toxicity study (90-day feeding test)

#### 5.1. Materials and animals

Lactase powder with a purity of 83.33% was used in this test. Eighty 5-week-old SD rats (40 male and 40 female) were sacrificed in this study. The lactase was incorporated into the rodent basic diet at concentrations of 16.49, 164.9 and 1649 mg/kg, respectively, which represents concentrations that were 10, 100 and 1000-fold greater than the mean human exposure. The diets were prepared one week before the test and stored at 4°C until use. The activity of lactase was maintained during the test.

#### 5.2. Experimental design

Following one week of acclimatization, rats of each gender were randomly divided into four groups (10/sex/group). Three experimental groups were fed with diets containing lactase at 16.49, 164.9 and 1649 mg/kg, the maximum dose in accordance with 1000-fold greater than the mean human exposure, and a control group was fed with the rodent basic diet. During the exposure time, body weight and food consumption were determined weekly. Food efficiency was calculated from food consumption and body weight. Feed utilization (%) = (body weight gain/feed consumption)×100. Detailed observations, including but not limited to changes in coat condition, skin, eyes, excretions, mentality, mortality and other clinical signs of toxicity, were recorded once a day throughout the 90-day feeding study.

Hematology and serum biochemistry values were measured on days 45 and 90. Blood was collected from the orbital sinus under anesthesia after fasting overnight (16 h). The blood samples used for hematology evaluation were collected in tubes with the anticoagulant ethylenediaminetetraacetic acid-2K (EDTA·K_2_), and they were analyzed to obtain white blood cell count (WBC), red blood cell count (RBC), hemoglobin (HGB), hematocrit (HCT), mean corpuscular volume (MCV), mean corpuscular hemoglobin (MCH), mean corpuscular hemoglobin concentration (MCHC), red cell volume distribution (RDW), blood platelet count (PLT), and mean platelet volume (MPV) using a HEMAVET 950FS animal blood cell counter (Drew Scientific, Inc., Dallas, TX, USA).

Samples used for serum chemistry evaluation were centrifuged at 4000×*g* for 10 min, and the supernatant serum was analyzed to determine the amounts of alanine aminotransferase (ALT), total protein (TP), albumin (ALB), alkaline phosphatase (ALP), aspartate amino transferase (AST), glucose (GLU), blood urea nitrogen (BUN), creatinine (CREA), calcium (Ca), phosphorus (P), total cholesterol (CHO), triglycerides (TG), and lactate dehydrogenase (LDH) with an automatic Biochemical Analyzer 7020 (HITACHI, Tokyo, Japan).

On day 90, the rats were killed by decapitation under anesthesia. A complete gross necropsy was conducted on the viscera. Organs were trimmed of extraneous fat and weighed (paired organs weighed together), including heart, liver, lung (with bronchi), spleen, kidneys, adrenal glands, brain, thymus, ovaries (female), and testes (male). All of the weighed organs and portions of the stomach, intestine (duodenum, jejunum, ileum), uterus (female), and epididymides (male) were added to fixative. For the histopathological analysis, organs sections were fixed in 10% neutral buffered formalin, dehydrated, and embedded in paraffin. Paraffin sections (5 µm) were cut and mounted onto glass sides. All tissue sections were stained with hematoxylin and eosin (H&E) and examined under an optical microscope by pathologists at the Medicinal Plant Research Institute of the Chinese Academy of Medical Science (Beijing, China).

### 6. Statistical analysis

All data are described as the mean value ± standard deviation. Statistical analysis was performed by one-way analysis of variance (ANOVA) using the SPSS 13.0 software. Quantitative and categorical data for the male and female rats were analyzed by gender. Response variables of the GM groups were compared to the positive/negative groups separately. The significance level was set at *P*<0.05.

## Results

### 1. Acute oral toxicity study

None of the rats died during the observation period. No clinical signs of toxicity, such as ill health or abnormal behavior, occurred during the study. There were no abnormal observations in body weight of rats as shown in Table1. No abnormalities or pathological changes were observed in the necropsy. Under the experimental conditions, the maximum tolerated dose of lactase was greater than 30 mL/kg·BW. The lactase is incorporated into milk with a ratio of 1∶1000. So the milk consumption in this acute oral toxicity study is in accordance with 1500 L milk consumption of a 50 kg human.

**Table 1:** Body weight of rat in acute oral toxicity study.

Animal	Sex	Body weight of day 0	Body weight of day 7	Body weight of day 15
Rats	Male	194.7±2.9	257.9±9.2	306.7±7.7
	Female	187.9±5.3	199.3±9.4	220.0±7.0

### 2. Ames test

The results of the Ames test are shown in Table 2. The numbers of reverse mutation colonies for the positive control substances such as sodium azide (NaN_3_), 2-acetamidofluorene (2-AF), 1,8-dihydroxyanthraquinone (1,8-HAQ) and Nexon were at least 2-fold greater than the negative control. Recombinant lactase did not increase the number of revertants in the four *Salmonella* strains compared with their negative controls, regardless of the presence or absence of S9 mix. Additionally, no dose-dependent mutagenic effects were caused by the lactase. Lactase did not show any mutagenic activity under the experimental conditions.

**Table 2:** Result of Ames treated with lactase (n = 3).

Group	Dose	TA97		TA98		TA100		TA102	
	µg/plate	−S9	+S9	−S9	+S9	−S9	+S9	−S9	+S9
Negative control	0	135±12.6	172±11.5	40±3.5	38±6.0	171±10.0	172±7.5	239±18.2	278±11.5
	312.5	125±7.5	181±9.1	44±9.1	38±8.1	175±14.5	186±9.3	239±3.1	227±9.5
	625	133±11.4	162±7.1	38±11.7	34±3.6	172±6.4	186±10.4	232±7.1	238±10.0
lactase	1250	122±13.9	153±7.6	42±4.9	49±9.5	172±14.7	180±13.0	241±7.5	233±5.6
	2500	133±8.9	163±5.1	45±6.4	39±6.4	184±8.9	190±11.2	223±12.5	229±13.6
	5000	114±12.6	127±9.6	40±6.8	37±8.2	182±11.2	182±12.9	224±14.6	227±11.4
NaN3	1.5					>2000			
1,8-HAQ	50								1437±156.0
Nexon	50	>2000		1754±94.3				>2000	
2-AF	10		1437±125.3		>2000		1105±111.1		>2000

### 3. Bone marrow cell micronucleus test

The micronucleus incidence rate of the positive control group treated with 40 mg/kg·BW of cyclophosphamide was significantly higher (*P*<0.01) than that of the negative control group. The micronucleus incidence rate of the experimental groups treated with 312.5, 625 and 1250 mg/kg·BW lactase showed no statistically significant differences compared to the negative control group (*P*>0.05) (Table 3). Additionally, no dose-dependent genetic toxicity was caused by the lactase. No genetic toxicity was observed for lactase under the experimental conditions.

**Table 3:** Effect of bone marrow cell micronucleus treated with lactase (*n* = 5).

Group		Male			Female		
	Dose (mg/kg)	Totalnumber	Number ofmicronucleus	Micronucleusrate(‰)	Totalnumber	Number ofmicronucleus	Micronucleusrate(‰)
Negative control	0	5×1000	28	5.5±1.4	5×1000	27	5.3±1.3
lactase	312.5	5×1000	34	6.8±1.4	5×1000	27	5.4±0.8
	625	5×1000	30	6.0±1.6	5×1000	28	5.6±1.2
	1250	5×1000	31	6.2±1.4	5×1000	37	7.4±2.8
Positive control	40	5×1000	132	26.4±2.0*	5×1000	109	21.8±3.4*

*: *p<0.01* compared with the negative control.

### 4. Mouse sperm abnormality test

The rate of sperm abnormalities in the positive control group was statistically higher than that of the negative group (*P*<0.01). The experimental groups treated with difference doses of lactase were all within the normal range, and there were no statistically significant differences in the lactase-treated groups compared to the negative control group (*P*>0.05) (Table 4). No mutagenic activity was observed under the experimental conditions.

**Table 4:** Mice sperm abnormality rate treat with lactase (n = 5).

	Dose(mg/kg)	totalnumber	Number ofabnormality	Rate ofabnormality(%)
Negative control	0	5×1000	71	1.42±0.36
	312.5	5×1000	85	1.70±0.32
lactase	625	5×1000	82	1.64±0.23
	1250	5×1000	99	1.98±0.42
Positive control	40	5×1000	244	4.88±0.51*

*: *P<0.01* compared with the negative control.

### 5. Ninety-day feeding test in rats

#### 5.1 Lactase activity in the diet during the 90-day feeding period

The activity of lactase in the feed was recorded at the beginning and after storage for one, two or three months, as shown in Table 5. The processing of the feed did not significantly influence the activity of lactase; the preservation rate was approximately 95%. After storage for three months, the activity of lactase remained at 90%.

**Table 5:** The lactase enzyme activity in feed (n = 3).

Group	Theconcentrationof lactase	Initiallactaseactivity	Lactaseactivityof feed	Preservationrate	Lactaseactivityafter onemonth	Preservationrate aftera month	Lactaseactivityafter twomonth	Preservationrate aftertwo month	Lactaseactivityafter threemonth	Preservationrate afterthree month
	(mg/kg)	(U/g)	(U/g)	(%)	(U/g)	(%)	(U/g)	(%)	(U/g)	(%)
Low-dose group	16.49	9.728±0.36	9.194±0.16	94.51±1.68	9.127±0.14	93.82±1.40	9.102±0.15	93.56±1.56	9.023±0.13	92.75±1.32
Mid-dose group	164.9	97.69±2.14	93.97±1.83	96.19±1.88	91.39±1.02	93.55±1.05	89.84±0.85	91.96±0.87	88.26±0.57	90.35±0.58
High-dose group	1649	978.3±5.32	928.2±10.3	94.88±1.05	910.3±6.35	93.05±0.65	903.2±8.34	92.32±0.85	894.3±6.87	91.41±0.70

#### 52. Mortality and clinical observations

**Figure 1:**
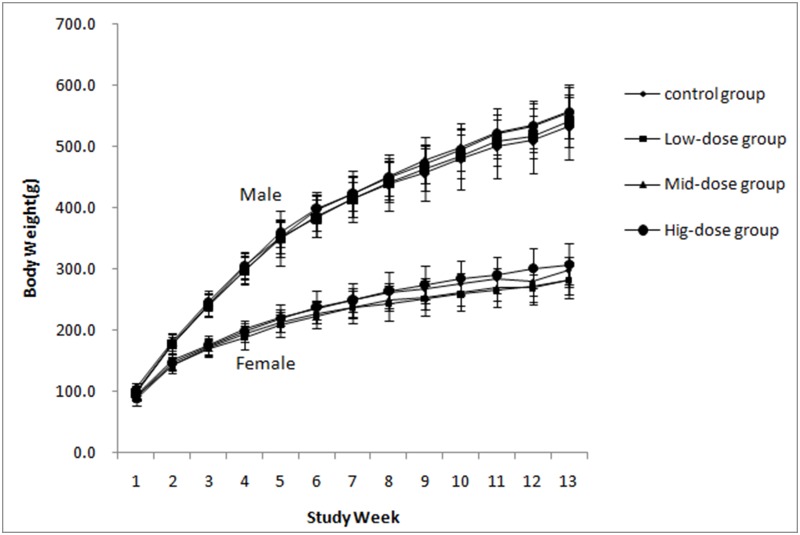
Mean weekly body weight of rats.

No deaths occurred during the 90-day feeding trial. No clinical signs of toxicity were observed, such as ill health or abnormal behavior. Body weight changes (Fig. 1, Table 6) and food consumption (Table 6) were comparable among animals of the same sex, regardless of lactase treatment.

**Table 6:** Food consumption and body weight of 90-day feeding.

Sex/Group	Ck	Low-dosegroup	Mid-dosegroup	High-dosegroup
*Males*				
Starting weight(g)	101±7	99±11	106.6±7.3	101±13
Final weight(g)	533±53	541±40	557±44	556±41
Weight gains(g)	432±53	442±40	451±43	455±40
Food consumption(g)	1921±73	1868±30	1987±20*	1966±83
Food utilization(%)	22.5	23.6	22.7	23.1
*Females*				
Starting weight(g)	94.2±5.7	93.3±7.4	87.8±9.6	93.5±8.6
Final weight(g)	298±22	283±29	282±23	308±36
Weight gains(g)	204±20	189±25	194±21	214±31
Food consumption(g)	1255±46	1214±30	1175±57	1300±41
Food utilization(%)	16.2	15.6	16.6	16.5

*: *P*<0.05 compared with the negative control.

#### 5.3. Hematology

At the end of the trial, most of the hematology parameters showed no significant differences in males or females between the lactase-treated animals and the negative controls (Table 7). There were significant differences in some parameters, but these effects were not considered to be related to lactase treatment. In males, a statistically significant decrease in WBC was observed in the middle-dose group. There were no significant differences between the high-dose group and the negative control group, and no dose-dependent effects were found. Therefore, the differences were clearly not related to dietary exposure to recombinant lactase. In females, the value of MCH in the group the group that consumed low-dose lactase and the value of MCHC in the group that consumed mid-dose lactase were significantly lower than the values for the negative control group. Significant differences were not observed in the high-dose groups, and the observed changes were small (less than 3%). Therefore, these differences were not dose- or gender-related.

**Table 7:** Terminal blood hematology values of rats fed lactase for 90-days (mean ± SD, n = 10).

Group	Negative control	Low-dose group	Middle-dose group	High-dose group
*Males*				
WBC (*10^9^/L)	13.7±1.7	12.5±2.5	9.93±2.53*	12.4±2.6
RBC (*10^12^/L)	8.46±0.31	8.24±0.30	8.30±0.47	8.70±0.46
HGB (g/L)	150±6	146±5	146±6	156±7
PLT (*10^9^/L)	688±99	698±66	662±98	676±65
HCT (%)	47.2±2.2	45.7±1.4	45.8±2.1	48.7±1.8
MCV (fL)	55.8±1.7	55.6±1.8	55.2±1.3	55.8±1.7
MCH (pg)	17.8±0.3	17.7±0.7	17.7±0.5	17.9±0.6
MCHC (g/L)	319±5	319±4	320±3	320±5
MPV (fL)	7.50±0.30	7.60±0.20	7.60±0.30	7.50±0.30
*Females*				
WBC (*10^9^/L)	8.07±2.03	7.56±2.21	6.44±1.69	6.95±1.09
RBC (*10^12^/L)	7.55±0.46	7.85±0.31	7.54±0.33	7.67±0.37
HGB (g/L)	146±6	144±5	140±6	144±5
PLT (*10^9^/L)	659±60	640±77	713±68	660±109
HCT (%)	45.0±1.8	45.3±1.7	44.3±1.6	45.3±1.4
MCV (fL)	59.7±2.8	57.7±1.1	58.8±1.7	59.1±1.8
MCH (pg)	19.0±0.5	18.4±0.3*	18.6±0.4	18.8±0.5
MCHC (g/L)	323±7	318±6	317±4*	317±5
MPV (fL)	7.60±0.20	7.70±0.30	7.70±0.10	7.70±0.40

*: *P*<0.05 compared with the negative control.

#### 5.4. Serum chemistry

As shown in Table 8, a majority of serum chemistry parameters showed no significant differences in the male and female groups between those fed with different diets and those given the negative control treatment. However, statistically significant differences were observed in some parameters. For reasons described below, none of the statistically significant differences were considered to be related to the consumption of recombinant lactase. In males, the mean AST and TP values in the mid-dose group were somewhat lower (*P<*0.05) than those of the negative control group. These significant differences were not considered to be biologically meaningful because no significant difference was observed in the high-dose group. The mean BUN value of the low-dose group was significantly different from that of the negative control group, but there were no significant differences in the mid- or high-dose groups. There were no dose- or gender-related effects among the differences. In females, the mean P value in the mid-dose group was lower than that of the negative control group. This difference was not considered to be biologically meaningful because no dose- or gender-related effects were observed.

**Table 8:** Terminal serum chemistry values of rats fed lactase for 90-days (mean ± SD, n = 10).

Group	Negative control	Low-dose group	Middle-dose group	High-dose group
*Males*				
LDH (U/L)	2296±558	2155±433	1882±516	2046±781
ALT (U/L)	35.7±7.7	30.3±4.2	30.2±9.4	32.0±5.5
AST (U/L)	178±16	162±24	158±22*	175±52
ALP (U/L)	136±39	121±14	127±28	118±29
TP (g/L)	75.8±3.3	73.6±1.9	72.1±3.2*	74.9±3.8
ALB (g/L)	37.5±0.7	37.2±1.8	37.9±0.9	38.1±0.6
BUN (mmol/L)	3.58±0.15	3.36±0.16*	3.76±0.92	3.62±0.37
CREA (umol/L)	66.8±3.9	63.8±2.3	64.8±9.2	63.6±3.9
GLU (mmol/L)	5.16±1.13	5.75±0.98	5.95±1.23	5.69±0.66
CHO (mmol/L)	1.48±0.41	1.47±0.16	1.46±0.14	1.54±0.26
TG (mmol/L)	0.70±0.36	0.59±0.19	0.72±0.34	0.83±0.68
Ca (mmol/L)	4.46±0.32	4.28±0.18	4.26±0.20	4.35±0.24
P (mmol/L)	2.30±0.20	2.18±0.08	2.15±0.16	2.20±0.08
*Females*				
LDH (U/L)	1742±649	1398±483	1753±412	1564±475
ALT (U/L)	38.7±12.8	32.7±8.7	35.8±11.0	32.6±6.0
AST (U/L)	149±28	147±35	154±28	151±26
ALP (U/L)	69.1±14.7	69.3±17.0	79.8±35.9	79.4±29.8
TP (g/L)	81.9±5.2	84.6±3.1	82.5±4.7	77.3±5.0
ALB (g/L)	47.2±3.0	46.7±2.6	47.2±2.2	46.8±2.6
BUN (mmol/L)	5.00±1.33	4.21±0.66	4.10±0.88	4.04±0.80
CREA (umol/L)	76.1±9.4	74.7±6.8	73.8±8.3	70.0±3.2
GLU (mmol/L)	5.04±0.87	5.48±0.70	5.63±0.49	5.16±1.00
CHO (mmol/L)	1.76±0.53	1.96±0.21	1.79±0.28	1.80±0.47
TG (mmol/L)	0.57±0.18	0.67±0.32	0.50±0.24	0.48±0.22
Ca (mmol/L)	4.76±0.22	4.81±0.25	4.53±0.29	4.35±0.30
P (mmol/L)	1.95±0.14	1.90±0.15	1.75±0.12*	1.89±0.15

*: *P*<0.05 compared with the negative control.

#### 5.5. Organ weights

No statistically significant differences in organ/body weight ratios (‰) were observed between the groups consuming different doses of recombinant lactase and the group that received only the basic diet (Table 9).

**Table 9:** Organ/body weight ratio(‰) of males and females (mean ± SD, n = 10).

Group	Negative control	Low-dose group	Middle-dose group	High-dose group
*Males*				
Heart	2.85±0.37	2.76±0.28	3.09±0.68	2.87±0.29
Liver	30.49±7.06	26.27±1.77	33.21±4.78	31.91±4.94
Spleen	1.51±0.35	1.33±0.18	1.39±0.19	1.27±0.51
Lung	3.77±0.73	3.96±0.63	3.68±0.88	4.02±0.84
Kidney	6.39±1.26	5.74±0.23	6.14±0.51	6.07±0.84
Brain	3.64±1.47	3.50±0.56	3.44±0.42	3.47±0.30
Adrenal gland	0.10±0.03	0.09±0.03	0.09±0.02	0.09±0.03
Thymus	0.82±0.19	0.83±0.18	0.79±0.22	0.87±0.18
Spermary	6.28±0.89	5.82±0.50	6.09±0.60	5.89±0.71
*Females*				
Heart	3.07±0.30	3.07±0.43	3.53±0.96	3.05±0.21
Liver	31.18±4.37	29.69±3.31	32.20±4.25	31.65±3.04
Spleen	1.40±0.22	1.42±0.27	1.61±0.25	1.51±0.19
Lung	4.31±0.75	4.64±0.95	4.93±0.86	4.99±0.81
Kidney	6.50±0.83	5.87±0.67	6.35±0.41	6.08±0.41
Brain	6.17±0.49	5.63±0.65	6.08±1.11	5.52±0.87
Adrenal gland	0.20±0.02	0.19±0.02	0.22±0.03	0.22±0.05
Thymus	1.12±0.25	1.03±0.33	1.12±0.27	1.04±0.35
Ovary	0.41±0.10	0.40±0.10	0.45±0.10	0.38±0.09

*: *P*<0.05 compared with the negative control.

#### 5.6. Gross necropsy and histopathology

No macroscopic pathological changes were observed in the necropsy and no treatment-related histopathological observations. All microscopic changes that were observed, including aspiration pneumonia, fatty liver, renal cysts and pyelectasin in the kidney, were types of changes that are commonly observed in rats. These changes also occurred in the negative control group, so they did not appear to have toxicological meaning in this study.

## Discussion

To satisfy the safety requirements for food additives, recombinant lactase was subjected to a series of toxicological tests in this study. In the acute test, the maximum tolerated dose of lactase was greater than 30 mL/kg·BW, which is in accordance with the 1500 L milk consumption of a 50 kg human. This is about 5400-fold greater than the human daily consumption. It was demonstrated that the LD_50_ of recombinant lactase is greater than 30 mL/kg·BW, according to the results of the acute oral toxicity test. Histidine-requiring auxotroph strains of *Salmonella Typhimurium* were used to test for potential mutagenic activity. The bacterial reverse mutation assay indicated no cytotoxic or mutagenic effects. The *in vivo* bone micronucleus test and sperm abnormality test showed no abnormalities in any of the parameters. In the 90-day feeding study, the administration of recombinant lactase at dosages of 16.49 mg/kg, 164.9 mg/kg, and 1649 mg/kg in the diet did not produce any significant treatment-related toxicological effects in terms of body weight, food consumption, organ weights, hematological parameters, clinical chemistry and histopathology compared to the control groups. Slight statistically significant differences were found in some of the hematological parameters compared to control group, but these differences did not appear to be treatment-related. Similarly, the statistically significant differences that occurred in certain clinical chemistry parameters were not treatment-related; they were not caused by lactase. Such differences are often found in other 90-day feeding tests, but they have no toxicological significance [28], [29], [30]. It should be noted that the doses were approximately 10-, 100- and 1000-fold greater than human daily exposure. The toxicological evaluations provided considerable evidence of safety because the tested dosages are much greater than daily consumption. This lactase showed no acute, mutagenic, genetic and subchronic toxicity under our study conditions.

Microorganisms are used in many industrial processes, such as the production of enzymes, vitamins, polysaccharides, polyhydric alcohols and lipids [31]. Recombinant DNA technology has made it possible to manufacture novel enzymes with high production efficiency, even when such production would be inefficient using traditional methods. The industrial production of enzymes for use in food additives dates to 1874, when Hansen extracted chymosin from calf stomachs for use in cheese production [32]. The US Food and Drug Administration (FDA) determined that lactase preparations from *Kluyveromyces lactis* could be generally recognized as safe in 1984. In 1991, bovine chymosin expressed in *Escherichia coli* K-12 became the first recombinant enzyme approved for use in food by the FDA [33]. The microbial enzymes that are used in food processing are normally called *enzyme preparations,* and many enzymes used in food processing are listed in the Pariza and Johnson review [34]. The Enzyme Technical Association (ETA) periodically updates the enzymes list and maintains it on their web site (http://www.enzymetechnicalassoc.org/). Based on various safety and toxicology evaluations, *P. pastoris* fulfills the criteria of a non-toxic and non-pathogenic microorganism [34], [35]. *P. pastoris* has been safely used for the production of over 300 recombinant proteins since the mid-1980s [36], and extensive reviews of databases have failed to identify the toxic effects caused by *P. pastoris*.

There is growing concern about food additives and dietary supplements. One juice was found not to be mutagenic, clastogenic, cytotoxic, or genotoxic, as determined by safety evaluation methods same as my research. The no-observed-adverse-effect level (NOEAL) was determined to be 40 g/kg·BW/day for male and female rats [28]. Coenen reported a lactase enzyme preparation derived from *Kluyveromyces lactis* that had a one-day NOAEL in the acute toxicity study under 10,000 mg/kg body weight, and the preparation did not induce noticeable signs of toxicity at that dosage, even after 28 days of feeding [37].

Microbial *enzyme preparations* must be food grade and meet applicable regulatory standards. The toxicological potential evaluation should be the primary consideration in evaluating enzyme safety. *In vivo* and *in vitro* tests are available for the detection of toxicity. Pariza and Foster (1983) regarded it was needed for testing new food *enzyme preparations* for mutagenic activity [34], and Ames test has been widely employed to indicate whether a given chemical may cause cancer *in vitro*. We considered four animal toxicity tests to be appropriate for evaluating the safety of this enzyme, an acute oral toxicity, bone marrow cell micronucleus test, mouse sperm abnormality test and 90-day oral subchromic study, all are conducted using the oral/diet route of administration as this is the intended exposure route of consumers. This toxicological evaluation system is helpful and can be used in the safety evaluation of other enzyme.

In the experiments presented here, the quantity of lactase administered to the rats is equivalent to what would be ingested by a 60-kg adult consuming 3.36×10^5 ^g of milk or dairy products per day. The data acquired in this research indicate that there are no safety concerns for using this lactase preparation from *P. pastoris* in the production of dairy products.
